# Supplementary material for: From sequence to enzyme mechanism using multi-label machine learning
Source: BMC Bioinformatics. 2014 May 19;15:150. doi: 10.1186/1471-2105-15-150 (PMC4229970; doi:10.1186/1471-2105-15-150)
Supplement: Additional file 2 — Java code of ml2db. Additional file ml2db_code.tar.gz contains the Java source code to run the multi-label machine learning experiments and save the results to database. The code’s Javadoc is included. [file 1471-2105-15-150-S2.zip › additional file 2/ml2db/utils/doc/index-files/index-3.html]

C-Index


JavaScript is disabled on your browser.


- Overview
- Package
- Class
- Use
- Tree
- Deprecated
- Index
- Help

- Prev Letter
- Next Letter

- Frames
- No Frames

- All Classes

A B C D E F G H I J K L M N O P Q R S T U V W X Y 


## C

calcAverage(double[]) - Static method in class uk.ac.standrews.utils.main.Utils
:   Calculates the average of the numbers in an array (from web
    http://forum.java.sun.com/, author JosAH)

calcStandardDeviation(double[]) - Static method in class uk.ac.standrews.utils.main.Utils
:   Calculates the standard deviation of the elements in an array as root of
    the variance = [sum( x(i) - mean)^2 )] / (n-1), where x(i) is the ith
    element, m the mean, n the number of elements

canReadFile(File) - Static method in class uk.ac.standrews.utils.main.FileUtils
:   Tests wether the file can be read

canReadFile(String) - Static method in class uk.ac.standrews.utils.main.FileUtils
:   Tests whether the file can be read

canReadParent(String) - Static method in class uk.ac.standrews.utils.main.FileUtils
:   Checks whether a file directory is readable.

canWriteFile(File) - Static method in class uk.ac.standrews.utils.main.FileUtils
:   Tests whether the file can be written

canWriteFile(String) - Static method in class uk.ac.standrews.utils.main.FileUtils
:   Tests wether the file can be written

canWriteParent(String) - Static method in class uk.ac.standrews.utils.main.FileUtils
:   Checks whether a file directory is writable.

cdf(double) - Method in class cern.jet.random.Pareto
:   Returns the cumulative distribution function.

cern.colt - package cern.colt


cern.colt.function - package cern.colt.function


cern.jet.random - package cern.jet.random


cern.jet.random.engine - package cern.jet.random.engine


CHAR\_2\_SQL\_DATATYPE - Static variable in class uk.ac.standrews.utils.main.database.DbUtils


CHAR\_4\_SQL\_DATATYPE - Static variable in class uk.ac.standrews.utils.main.database.DbUtils


CHAR\_6\_SQL\_DATATYPE - Static variable in class uk.ac.standrews.utils.main.database.DbUtils


checkRow() - Method in class uk.ac.standrews.utils.main.database.TableRow


checkRowFields() - Method in class uk.ac.standrews.utils.main.database.TableRow
:   Checks that the record values are paired to fields existing in the table.

checkRowValues() - Method in class uk.ac.standrews.utils.main.database.TableRow
:   TODO Not implemented: check that each row value is of the appropriate
    datatype for the table field...

choiceEvent - Variable in class uk.ac.standrews.utils.main.guiutils.SimpleRadioButtonPanel


choose(int) - Method in class edu.cornell.lassp.houle.RngPack.RandomElement


choose(int, int) - Method in class edu.cornell.lassp.houle.RngPack.RandomElement


chopper(String) - Static method in class uk.ac.standrews.utils.main.Scripts


classFieldNames(Object) - Static method in class uk.ac.standrews.utils.main.ReflectionUtils
:   public static String classFieldsNamesAndValuesString(Object o){
    TreeMap map =
    ReflectionUtils.classFieldsNamesAndValues(o); String fieldsValues =
    "Fields of " + o.getClass().getName() + "\n"; Set>
    entries = map.entrySet(); Iterator> it =
    entries.iterator(); while(it.hasNext()){ Entry entry =
    it.next(); fieldsValues += entry.getKey() }
    return fieldsValues;
    }

classFieldsNamesAndValues(Object) - Static method in class uk.ac.standrews.utils.main.ReflectionUtils


cleanSet(Set<String>) - Static method in class uk.ac.standrews.utils.main.collectionutils.ListUtils
:   Cleans a set from null and empty elements

clear() - Method in class uk.ac.standrews.utils.main.maputils.OneToManyMap


ClientHttpRequestUtils - Class in uk.ac.standrews.utils.main.webutils
:   Title: Client HTTP Request class

ClientHttpRequestUtils(String) - Constructor for class uk.ac.standrews.utils.main.webutils.ClientHttpRequestUtils
:   Creates a new multipart POST HTTP request for a specified URL string

ClientHttpRequestUtils(URL) - Constructor for class uk.ac.standrews.utils.main.webutils.ClientHttpRequestUtils
:   Creates a new multipart POST HTTP request for a specified URL

ClientHttpRequestUtils(URLConnection) - Constructor for class uk.ac.standrews.utils.main.webutils.ClientHttpRequestUtils
:   Creates a new multipart POST HTTP request on a freshly opened
    URLConnection

ClockSeed() - Static method in class edu.cornell.lassp.houle.RngPack.RandomSeedable
:   Return a long integer seed calculated from the date.

ClockSeed(Date) - Static method in class edu.cornell.lassp.houle.RngPack.RandomSeedable
:   Return a long integer seed given a date

clone() - Method in class cern.colt.PersistentObject
:   Returns a copy of the receiver.

clone() - Method in class cern.jet.random.AbstractDistribution
:   Returns a deep copy of the receiver; the copy will produce identical
    sequences.

clone() - Method in class cern.jet.random.engine.MersenneTwister
:   Returns a copy of the receiver; the copy will produce identical
    sequences.

clone() - Method in class edu.cornell.lassp.houle.RngPack.RandomShuffle
:   Returns a copy of the receiver.

clone() - Method in class edu.cornell.lassp.houle.RngPack.Ranlux
:   Returns a copy of the receiver; the copy will produce identical
    sequences.

clone() - Method in class edu.cornell.lassp.houle.RngPack.Ranmar
:   Returns a copy of the receiver; the copy will produce identical
    sequences.

clone() - Method in class uk.ac.standrews.utils.main.collectionutils.setutils.Set


clone() - Method in class uk.ac.standrews.utils.main.setutils.Set


closeConnection() - Method in class uk.ac.standrews.utils.main.database.DbConn
:   Close the database connection

closeConnection() - Method in class uk.ac.standrews.utils.main.database.DbManager


CollectionUtils - Class in uk.ac.standrews.utils.main.collectionutils
:   Class

CollectionUtils() - Constructor for class uk.ac.standrews.utils.main.collectionutils.CollectionUtils


CollectionUtilsTest - Class in uk.ac.standrews.utils.test
:   Class

CollectionUtilsTest() - Constructor for class uk.ac.standrews.utils.test.CollectionUtilsTest


columnExistsInTable(String) - Method in class uk.ac.standrews.utils.main.database.TableReader


CommandLineMenu - Class in uk.ac.standrews.utils.main.guiutils
:   Stores the options for a command line program.

CommandLineMenu(String, String, ArrayList<CommandOption>, String, String) - Constructor for class uk.ac.standrews.utils.main.guiutils.CommandLineMenu


CommandOption - Class in uk.ac.standrews.utils.main.guiutils


CommandOption(String, String) - Constructor for class uk.ac.standrews.utils.main.guiutils.CommandOption


compare(TableColumn, TableColumn) - Method in class uk.ac.standrews.utils.main.database.TableColumn
:   Compares two columns by their name, returns -1 if f1 comes alphabetically
    before f2, 0 if the two are equal and +1 if f2 comes alphabetically
    before f1.

compareTo(Set) - Method in class uk.ac.standrews.utils.main.collectionutils.setutils.Set


compareTo(TableColumn) - Method in class uk.ac.standrews.utils.main.database.TableColumn
:   Compares two columns by their name, returns -1 if this column comes
    alphabetically before f2, 0 if the two are equal and +1 if f2 comes
    alphabetically before this column.

compareTo(Set) - Method in class uk.ac.standrews.utils.main.setutils.Set


connectionIsValid(Connection) - Static method in class uk.ac.standrews.utils.main.database.DbConn
:   Checks that the connection is not null

connectionIsValid() - Method in class uk.ac.standrews.utils.main.database.DbConn


connectionIsValid() - Method in class uk.ac.standrews.utils.main.database.DbManager


contains(Set) - Method in class uk.ac.standrews.utils.main.collectionutils.setutils.Set


contains(Set) - Method in class uk.ac.standrews.utils.main.setutils.Set


containsInternalKeyValue(String, String) - Method in class uk.ac.standrews.utils.main.maputils.MapOfMaps


containsKey(Object) - Method in class uk.ac.standrews.utils.main.maputils.OneToManyMap
:   Checks if the map contains a key

containsKey(String) - Method in class uk.ac.standrews.utils.main.maputils.TableMap


containsKeyKeyValue(String, String, String) - Method in class uk.ac.standrews.utils.main.maputils.MapOfMaps
:   Checks if the map contains a specific [external key, internal key, value]
    triple

containsKeyValue(T, U) - Method in class uk.ac.standrews.utils.main.maputils.OneToManyMap
:   Checks if the map contains a specific key-value pair

containsKeyValue(String, String) - Method in class uk.ac.standrews.utils.main.maputils.TableMap


containsPositiveInteger(String) - Static method in class uk.ac.standrews.utils.main.stringutils.StringUtils
:   Returns true if the string contains a parsable integer

containsValue(Object) - Method in class uk.ac.standrews.utils.main.maputils.OneToManyMap
:   Checks if the map contains a value

containsValue(String) - Method in class uk.ac.standrews.utils.main.maputils.TableMap


countInternalKeys() - Method in class uk.ac.standrews.utils.main.maputils.MapOfMaps
:   Get how many unique internal keys are there in all the internal maps

countValues(Collection, Object) - Static method in class uk.ac.standrews.utils.main.collectionutils.CollectionUtils
:   Counts the occurrences of a given value in a collection (using the
    'equals' method)

createSupersets() - Method in class uk.ac.standrews.utils.main.collectionutils.setutils.SupersetsManager


createSupersets() - Method in class uk.ac.standrews.utils.main.setutils.SupersetsManager


createTable() - Method in class uk.ac.standrews.utils.main.database.TableCreator
:   Creates the database table for this `Table` object

createTables() - Method in class uk.ac.standrews.utils.main.database.DbCreator

A B C D E F G H I J K L M N O P Q R S T U V W X Y

- Overview
- Package
- Class
- Use
- Tree
- Deprecated
- Index
- Help

- Prev Letter
- Next Letter

- Frames
- No Frames

- All Classes
